# Polymorphisms of the DNA Methyltransferase 1 Associated with Reduced Risks of *Helicobacter pylori* Infection and Increased Risks of Gastric Atrophy

**DOI:** 10.1371/journal.pone.0046058

**Published:** 2012-09-25

**Authors:** Jing Jiang, Zhifang Jia, Donghui Cao, Mei-Shan Jin, Fei Kong, Jian Suo, Xueyuan Cao

**Affiliations:** 1 Division of Clinical Epidemiology, Jilin University First Hospital, Changchun, China; 2 Division of Pathology, Jilin University First Hospital, Changchun, China; 3 Department of Gastric and Colorectal Surgery, Jilin University First Hospital, Changchun, China; National Cancer Center, Japan

## Abstract

**Introduction:**

DNA methyltransferase-1(*DNMT1*) is an important enzyme in determining genomic methylation patterns in mammalian cells. We investigated the associations between SNPs in the *DNMT1* gene and risks of developing *H. pylori* seropositivity, gastric atrophy and gastric cancer in the Chinese population.

**Methods:**

The study consisted of 447 patients with gastric cancer; 111 patients with gastric atrophy; and 961 healthy controls. Five SNPs, rs10420321, rs16999593, rs8101866, rs8111085 and rs2288349 of the *DNMT1* gene were genotyped. Anti-*H*.*pylori* IgG was detected by ELISA. Gastric atrophy was screened by the level of serum pepsinogen Ιand II and then confirmed by endoscopy and histopatholgical examinations.

**Results:**

The age- and sex-adjusted OR of *H*. *pylori* seropositivity was 0.67 (95%CI: 0.51–0.87) for rs8111085 TC/CC genotypes, significantly lower than the TT genotype in healthy controls. The adjusted OR of *H*.*pylori* seropositivity was 0.68 (95%CI: 0.52–0.89) for rs10420321 AG/GG genotypes. In addition, patients carrying rs2228349 AA genotype have a significantly increased risk for *H*.*pylori* seropositivity (OR = 1.67; 95%CI: 1.02–2.75). Further haplotype analyses also showed that the ATTTG and ATCTA are significantly associated with increased risks in *H*.*pylori* infection compared to the GTCCG haplotype (OR = 1.38, 95%CI: 1.08–1.77; OR = 1.40, 95% CI: 1.09–1.80). The adjusted ORs of gastric atrophy were 1.66 (95%CI: 1.06–2.61) for rs10420321 GG genotype, and 1.67 (95%CI 1.06–2.63, P = 0.03) for rs8111085 CC genotype, but no association was found between SNPs in the *DNMT1* gene and risk of developing gastric cancer.

**Conclusions:**

Individuals with rs10420321 GG and rs8111085 CC genotype of the *DNMT1* gene were associated with reduced risks for *H.pylori* infection. On the other hand, higher risks of gastric atrophy were found in the carriers with these two genotypes compared to other genotypes. Our results suggested that SNPs of *DNMT1* could be used as genotypic markers for predicting genetic susceptibilities to *H*.*pylori* infection and risks in gastric atrophy.

## Introduction

Gastric cancer is the most common malignancy of gastrointestinal tract in East Asians, and the third most common cause of cancer-related deaths in China [1], [2]. *Helicobacter pylori* (*H*.*pylori*) infection has been found to be a major risk factor for gastric cancer, through induction of gastric atrophy and progression to precancerous lesions [3], [4]. Although *H*.*pylori* is estimated to be found in at least half of the world’s population, few develop to gastric adenocarcinoma. The extent of gastric damages induced by *H*.*pylori* infection seems to vary between one person to another, suggesting that the interaction between the host genetic traits and the bacterial virulence plays an important role in long-term outcomes of *H*.*pylori* infection [5], [6], [7]. Single nucleotide polymorphisms (SNPs) are most common forms of genetic traits which may contribute genetic susceptibilities to gastric carcinogenesis.

DNA methylation is important in transcription regulation and chromatin remodeling in mammalian cells [8]. Aberrant DNA methylation of CpG islands is a common epigenetic change found in gastric cancers and *H*.*pylori* infection has been shown to induce alterations of DNA methylation in gastric mucosae [9], [10]. Increasing evidence suggests that aberrant methylation in gastric mucosae creates a field for cancerization happening in the early and precancerous stages. However, those studies focus on the impacts of environment factors (*H*.*pylori*), not host factors, such as methyltransferase (DNMT), which may affect epigenetic regulation.

DNA methyltransferase-1 (DNMT1) catalyzes the DNA post-replication methylation and maintains DNA methylation patterns during cell divisions [11]. Several studies have found that DNMT1 is over-expressed in human cancers including gastric cancer, suggesting that it may be involved in tumorgenesis and tumor progression [12], [13]. The *DNMT1* gene is located on chromosome 19p13.2 with a total size of 62 kb and is constituted of 40 exons. Mutations in coding regions of the *DNMT1* have been reported in colorectal cancer, such as a single base deletion in exon 23 resulting in deletion of the whole catalytic domain; a point mutation in exon 35 resulting in an amino acid substitution in the catalytic domain [14]. The inactivation of *DNMT1* by mutations can cause a genome-wide alteration of the DNA methylation status.

Two SNPs rs2241531 and rs4804490 of the *DNMT1* gene have been identified to be associated with clearance of HBV infection in the Korean population [15]. Polymorphism rs75616428, a nonsynonymous SNP in exon 4 of the *DNMT1* was demonstrated to be weakly associated with an increased production of anti-SSB (La) antibody in systemic lupus erythematosus patients [16]. Furthermore, an association was reported between the haplotypes of *DNMT1* and sensitivities to exposure of bensopyrene diol epoxide, supporting involvements of these SNPs in protecting the cell from DNA damage and reducing the intrinsic susceptibility to cancer [17].

These results suggested that the genetic variants of the *DNMT1* gene may modulate susceptibilities to virus infection and cancer development. Therefore, the aim of this investigation is to assess associations between polymorphisms in the *DNMT1* gene with *H*.*pylori* infection and clinical outcomes of *H*.*pylori* infection within the Chinese population.

## Methods

### Study Populations

Four hundred and forty seven gastric cancer cases were selected from the Department of Gastric and Colorectal Surgery, First Hospital of Jilin University, between 2008 and 2010. All patients underwent tumor resections with histological confirmed gastric adenocarcinoma. Individuals with gastric atrophy and healthy controls were recruited from examinees in the health check-up centre of the same hospital from 2009 to 2010. In brief, a total of 1111 individuals without cancer history (654 males and 457 females, ages of 35 to 80 years old) participated in the study. The examinees were Han descent from the area of Changchun. 150 subjects were found to have gastric atrophy by serum PG examination and 111 of them were confirmed by biopsy and histopathological examinations; 39 subjects were excluded from the study as they either had been diagnosed pseudopositive for gastric atrophy (17 cases) or had rejected endoscopic examination (22 cases). The remaining examinees (961) were included in the control group. Informed consent was obtained from all patients and the study protocol was approved by the ethics committee of the first affiliated hospital, Jilin University.

### Tests for *H.pylori* Infection and Diagnosis of Gastric Atrophy

Serum immunoglobulin (Ig) G antibodies to *H*. *pylori* were detected using a kit for *H*. *pylori* -Ig G enzyme-linked immunosorbent assay (ELISA) (Biohit, Helsink, Finland). The antibody titers were quantified by optical density (OD) readings according to the manufacturer’s protocol and titres higher than the threshold value of 30EIU were considered as positive for *H*. *pylori* infection. Levels of Pepsinogen Ι and II (PG Ι and PG II) in serum were measured using ELISA kits (Biohit, Helsink, Finland). For gastric atrophy screening, cut-off points used in this study were <82.3 ng/ml for PG Ι and <6.05 for PGΙ/PG II ratio, as they had previously been validated against histological confirmatory studies for gastric atrophy [18]. The quality control samples in kits showed coefficients of variation of 4.5%, 4.3% and 4.7% for *H*.*pylori*, PG Ι and PG II, respectively. All gastric atrophy suspected cases, based on the serum screening, were confirmed by endoscopy, biopsy and histological examinations for final diagnosis.

### Selection of Tagging SNPs

The principal hypothesis underlying this study is that one or more common SNPs in the *DNMT1* gene are associated with risks for *H*.*pylori* infection, gastric atrophy and gastric cancer. Thus, the aim of SNP tagging is to identify a set of SNPs that efficiently tag all known SNPs. We postulate that such SNPs are also likely to tag any hitherto unidentified SNPs in the *DNMT1* gene. Haplotype tagging SNPs (htSNPs) were selected from Han Chinese data in the HapMap Project (06-02-2009 HapMap) using the SNPbrowser™ Software v4.0 to capture SNPs with a minimum minor allele frequency (MAF) of 0.05 with a pair-wise r square of 0.8 or greater [19]. There are 27 SNPs at minor allele frequencies >0.05 in the *DNMT1* gene in Chinese on HapMap, Three SNPs lie in coding regions, including non-synonymous SNPs rs16999593 (His97Arg) in exon 4, rs8111085 (Ile327Val) in exon 12 and a synonymous SNP rs2228611 in exon 17. 24 SNPs reside in introns of the *DNMT1* gene and 21 of them are shown in a complete linkage disequilibrium (LD) with Tag SNP (D′ = 1 and r^2^>0.8). The synonymous SNP rs2228611 was excluded from genotyping due to being in an absolute LD (D′ = 1 and r^2^ = 1) with rs8101866. Finally, five SNPs rs10420321, rs16999593, rs8101866, rs2288349 and rs8111085 were selected for a larger-scale genotyping.

### Genotyping

Genomic DNA from whole blood sample was extracted with an AxyPrep blood Genomic DNA extraction Kit (AP-MN-BL-GDNA-250, Axygen Biosciences, Union City, USA). Polymerase chain reactions (PCR) were carried out in a 5 ul per reaction on the genomic DNA (10 ng) using a TaqMan universal PCR master mix (Applied Biosystems). Forward, reverse primers, FAM and VIC labeled probes were designed by Applied Biosystems (ABI Assay-by-Designs). Sequences of primers and probes are available on request. Amplification conditions on BIO-RAD S1000TM thermal cyclers (Bio-Rad Laboratories, Hercules, California) were as follows: 1 cycle of 95°C for 10 min, followed by 40 cycles of 95°C for 15 s and 60°C for 1 min. The PCR products were genotyped on an ABI PRISM 7900 HT Sequence Detector in end-point mode using the Allelic Discrimination Sequence Detector Software V2.3 (Applied Biosystems). For the software to recognize genotypes, two non-template controls were included in each of 384-well plates. All samples were arrayed together in four 384-well plates, and the fifth plate contained eight duplicate samples from each of four plates to ensure the quality of genotyping (the concordance was >99% for all SNPs).

### Statistics Analysis

Deviations of genotype frequencies in controls from those expected under the Hardy-Weinberg equilibrium were assessed by a goodness-of-fit x^2^-test. Linkage disequilibrium (LD) between pairs of biallelic loci was determined using two measures, D´ and r^2^. Either Chi-square test or Fisher’s exact test was performed by comparing distributions of genotype frequencies between patients and controls. Risks associated with rare genotypes were estimated as odds ratios (ORs). Corresponding 95% confidence intervals (CIs) by unconditional logistic regression were adjusted by age (scale variable), sex (nominal variable) and *H*. *pylori* antibody (nominal variable). For haplotypes with frequencies >1%, risks were compared to those of the reference haplotype(major haplotype in the control group)using an unconditional logistic regression model with the HAPSTAT version 3.0 software (Tammy Bailey, Danyu Lin and the University of North Carolina, NC, USA) [20], [21]. All statistical tests were two-tailed and *P*-values less than 0.05 were considered to be statistically significant. All analyses were performed using statistical software for windows, SAS version 9.2 (SAS Institute, Cray, NC, USA). The power of the statistical tests was calculated using the QUANTO Version 1.2.3 software program (Jim Gauderman and John Morrison, University of Southern California, CA, USA).

## Results

### Allele Frequencies of the htSNPs

A total of 447 patients with gastric cancer (322 males and 125 females, aged between 35 to 80 years old), 111 subjects with gastric atrophy and 961 subjects who passed health checks were recruited in this study. The characteristics of subjects are summarized in table 1. The mean age was older in gastric cancer patients compared to the control group (61.6 vs. 50.6 years; *P*<0.001). There were more females in the control group (*P*<0.001). Prevalence rates of *H*.*pylori* seropositivity were significantly higher in the gastric cancer and gastric atrophy groups compared to the control group (69.1%, 75.7% vs. 49.7%, *P*<0.001). Genotype distributions for five SNPs in the control group were consistent with the Hardy-Weinberg equilibrium (*P* values were 0.32, 0.91, 0.31, 0.14 and 0.55 respectively). Rs10420321 and rs8111085 genotype distributions in the gastric atrophy group were found to be statistically significantly different to the control group and gastric cancer (*P* values were 0.04 and 0.05), however no statistically significant difference were found between groups for the remaining three SNPs.

**Table 1:** Characteristics of the subjects and the *DNMT*1 polymorphisms.

	ControlGroups n(%)	Gastric AtrophyGroups n(%)	Gastrc CancerGroups n(%)	*P* value
N	961	111	447	
Sex				
Male	564(58.7)	66(59.5)	322(72.0)	<0.001
Female	397(41.3)	45(40.5)	125(28.0)	
Age				
≤45	285(29.7)	20(18.0)	36(8.1)	<0.001
46–65	607(63.2)	81(73.0)	239(53.5)	
>65	69(7.2)	10(9.0)	172(38.5)	
*H.pylori*				
Negative	483(50.3)	27(24.3)	138(30.9)	<0.001
Postive	478(49.7)	84(75.7)	309(69.1)	
*DNMT*1				
rs10420321				
A/A	328(34.1)	32(28.8)	141(31.5)	0.04
A/G	454(47.2)	68(61.3)	230(51.5)	
G/G	179(18.6)	11(9.9)	76(17.0)	
rs16999593				
T/T	659(68.6)	78(70.3)	283(63.3)	0.07
T/C	273(28.4)	33(29.7)	144(32.2)	
C/C	29(3.0)	0	20(4.5)	
rs8101866				
G/G	489(50.9)	51(45.9)	238(53.5)	0.59
G/A	402(41.8)	49(44.1)	177(39.8)	
A/A	70(7.3)	11(9.9)	30(6.7)	
rs8111085				
T/T	330(34.3)	32(28.8)	143(32.0)	0.05
T/C	447(46.5)	67(60.4)	214(47.9)	
C/C	184(19.1)	12(10.8)	90(20.1)	
rs2288349				
G/G	515(53.6)	60(54.1)	249(56.1)	0.45
G/A	372(38.7)	47(42.3)	161(36.3)	
A/A	74(7.7)	4(3.6)	34(7.7)	

### Associations of SNPs with *H*.*pylori* Seropositivity, Gastric Atrophy and Gastric Cancer

Rates of *H*.*pylori* seropositivity were statistically significantly reduced in subjects bearing with rs8111085 TC and CC genotypes (*P* value = 0.01). The age-adjusted and sex-adjusted ORs relative to the TT genotype were 0.70 (95%CI: 0.52–0.93) and 0.60 (95%CI: 0.42–0.86), respectively, showing a dominant effect of the rare allele (OR = 0.67, 95%CI: 0.51–0.87). A similar association was found between rs10420321 and risk of *H.pylori* seropositivity, a dominant effect of the rare allele G. ORs were 0.62 (95%CI: 0.43–0.90) for the AG, 0.70 (95%CI: 0.53–0.94) for the GG genotype, and 0.68 (95%CI: 0.52–0.89) for the combined AG/GG. The heterogeneity test of association was not statistically significant for rs2228349 (*P* = 0.68), but evidence suggested a recessive mode of the rare allele (OR = 1.67; 95%CI: 1.02–2.75). Haplotypes with frequencies ≥1% are shown in table 2. Five major haplotypes accounted for over 98% of the distribution. Furthermore, the haplotype analysis revealed that the ATTTG and ATCTA were statistically significantly associated with increased risks for *H*.*pylori* seropositivity versus the GTCCG haplotype (OR = 1.38, 95%CI: 1.08–1.77; OR = 1.40, 95%CI: 1.09–1.80).

**Table 2:** *DNMT1* polymorphisms for *H.pylori* seropositivity in control groups.

Genotype	*H.pylori*(+)	*H.pylor*i(−)	OR(95%CI)*	*P* value
	(n = 478)(%)	(n = 483)(%)		
rs10420321				
A/A	38.5	29.8	Reference	
A/G	45.0	49.5	0.62(0.43–0.90)	0.01
G/G	16.5	20.7	0.70(0.53–0.94)	0.02
A/G+G/G	61.5	70.2	0.68(0.52–0.89)	0.01
rs16999593				
T/T	70.3	66.9	Reference	
T/C	27.0	29.8	0.87(0.66–1.16)	0.34
C/C	2.7	3.3	0.80(0.38–1.69)	0.55
rs8101866				
C/C	48.3	53.4	Reference	
C/T	43.9	39.8	1.25(0.76–2.07)	0.38
T/T	7.7	6.8	1.23(0.94–1.60)	0.13
rs8111085				
T/T	38.9	29.8	Reference	
T/C	44.4	48.7	0.70(0.52–0.93)	0.03
C/C	16.7	21.5	0.60(0.42–0.86)	0.01
T/C+C/C	61.1	70.2	0.67 (0.51–0.87)	0.01
rs2288349				
G/G	51.9	55.3	Reference	
G/A	38.7	38.7	1.06(0.81–1.38)	0.68
A/A	9.4	6.0	1.67(1.02–2.75)	0.04
Haplotpe				
GTCCG	22.5	27.4	Reference	
ATTTG	29.6	26.2	1.38(1.08–1.77)	0.01
ATCTA	28.6	25.1	1.40(1.09–1.80)	0.01
GCCCG	16.2	17.8	1.13(0.85–1.50)	0.39
ATCTG	2.7	2.4	1.41(0.78–2.54)	0.25

* OR were calculated by age and sex adjusted logistic regression model.

The age-, sex- and *H.pylori* antibody-adjusted ORs for gastric atrophy were 1.67 (95%CI 1.06–2.63) for the CC compared to the TT genotype at rs8111085 and 1.66 (95%CI: 1.06–2.61) for the GG versus the AA genotype at rs10420321. However, the haplotype analysis did not show association between the haplotype and the risk in gastric atrophy. Associations between the SNPs in the *DNMT1* gene and gastric cancer were also examined, but no significant correlation was found between the SNPs and gastric cancer risk (Table 3). In subgroup analysis, no correlation was found between the SNPs and Lauren’s classification, tumor differentiation and TNM staging (data not shown).

**Table 3:** *DNMT1* polymorphisms for gastric atrophy and gastric cancer.

Genotype	GA(%)	Control(%)	OR(95%CI)*	*P* value	GC(%)	Control(%)	OR(95%CI)*	*P* value
	n = 111	n = 961			n = 447	n = 961		
rs10420321								
A/A	28.8	34.1	Reference		31.5	34.1	Reference	
A/G	61.3	47.2	0.70(0.34–1.44)	0.34	51.5	47.2	0.96(0.66–1.41)	0.85
G/G	9.9	18.6	1.66(1.06–2.61)	0.03	17	18.6	1.17(0.88–1.55)	0.29
rs16999593								
T/T	70.3	68.6	Reference		63.3	68.6	Reference	
T/C	29.7	28.4	1.10(0.71–1.71)	0.66	32.2	28.4	1.25(0.95–1.66)	0.11
C/C	0.0	3.0			4.5	3	1.59(0.80–3.16)	0.18
rs8101866								
C/C	45.9	50.9	Reference		53.5	50.9	Reference	
C/T	44.1	41.8	1.46(0.71–2.96)	0.30	39.8	41.8	0.81(0.48–1.35)	0.42
T/T	9.9	7.3	1.16(0.76–1.77)	0.48	6.7	7.3	0.99(0.76–1.28)	0.91
rs8111085								
T/T	28.8	34.3	Reference		32	34.3	Reference	
T/C	60.4	46.5	0.76(0.38–1.52)	0.43	47.9	46.5	1.08(0.88–1.43)	0.61
C/C	10.8	19.1	1.67(1.06–2.63)	0.03	20.1	19.1	1.18(0.82–1.69)	0.38
rs2288349								
G/G	54.1	53.6	Reference		56.1	53.6	Reference	
G/A	42.3	38.7	1.03(0.68–1.55)	0.90	36.3	38.7	0.93(0.71–1.22)	0.93
A/A	3.6	7.7	0.41(0.14–1.16)	0.09	7.7	7.7	0.81(0.50–1.33)	0.81
Haplotpe								
ATTTG	32.0	28.0	Reference		26.7	28.0	Reference	
GTCCG	25.7	24.9	0.90(0.62–1.30)	0.57	22.8	24.9	0.90(0.73–1.14)	0.40
ATCTA	24.8	27.0	0.80(0.55–1.16)	0.25	25.2	27.0	1.00(0.77–1.19)	0.69
GCCCG	14.9	17.0	0.76(0.49–1.17)	0.22	19.4	17.0	1.20(0.92–1.48)	0.20
ATCTG	2.3	2.6	0.77(0.30–1.99)	0.58	3.8	2.6	1.50(0.93–2.37)	0.10

* OR were calculated by age, sex and H.pylori seropostivity adjusted logistic regression model.

## Discussion

Previous studies have shown that *H*.*pylori* infection could induce aberrant DNA methylation at the CpG islands, resulting in inactivations of tumor suppressor genes in gastric mucosa, and creating predisposed fields for cancerization. In the present study, we investigated impacts of host factors on *H*. *pylori* infection and carcinogenesis. The association between the SNPs of the *DNMT1* gene with *H*.*pylori* infection, gastric atrophy and gastric cancer in the Chinese Han population were studied. The results showed that the subjects bearing rs2228349 AA genotype has a significantly increased risk for *H*.*pylori* infection. In addition, our results revealed that C allele at rs8111085 and G allele at rs10420321 in the *DNMT1* gene protect the carrier against *H*.*pylori* infection, while, surprisingly, they are associated with increased risks in developing gastric atrophy.

Colonization of *H*.*pylori* at the human gastric mucosa is associated with complex local and systemic immune responses. In response to *H*.*pylori* infection, uncommitted naïve CD4+ T-cell precursors differentiate into several T helper (Th) cell subsets (Th1, Th2, Th17 and T regulatory cells) and secrete distinct cytokines. IFN-γ producing Th1 cells are important for the clearance of intracellular pathogens and IL-4 producing Th2 cells are crucial for humoral immunity and the clearance of extracellular pathogens [22]–[25]. Significant inverse correlations were found between bacterial colonization and gastric expressions of IFN-γ, TNF-α, IL-12 and IL-17 [26]. Increasing evidences showed that Th1 response, induced by *H*.*pylori* infection, contributes to the development of gastric atrophy [23]. In contrast, the Th2 cytokine IL-4 limits *H*.*pylori* associated gastric inflammation and favours *H*.*pylori* clearance [27]. Epigenetics are involved in the regulation of these developmental programs through activation or silencing of specifying genes during T-cell differentiation. IL-2, IL-3, IL-5, IL-10, IL-13, especially IL-4 and IFN-γ expressions were all up-regulated in CD4 and CD8 T cells with reduced DNA methylation, due to the lack of DNMT1 maintenance [28]–[32]. Therefore, we propose that the polymorphisms of *DNMT1* may impair the function of the DNMT1 protein, and fail to maintain the silenced state of Th1 signature genes in Th2 cells, skewing T-cell response towards Th1 responses, resulting in clearance of *H*.*pylori* infection and causing relative severe gastric atrophy at the same time.

So far, the functions of *DNMT1* polymorphisms are still unknown. The polymorphism rs8111085 at exon 12 leads to an amino acid change from Ile to Val, which may affect the corresponding DNMT1 protein structure or function. The protein may be impaired in its ability to stimulate de novo DNA methylation and to maintain the methylation status [33]. Rs10420321 and rs2288349 are both located within introns of *DNMT1*. Using bioinformatic tool (http://compbio.cs.queensu.ca/F-SNP/), rs10420321 on intron 1 is predicted to affect the transcriptional regulation of the DNMT1 mRNA, while rs2288349 does not appear to reside in any transcription factor binding sites or splicing sites [34]. Further LD analysis showed a complete LD (D′ = 0.996 and r^2^ = 0.985) between rs10420321 and the functional polymorphism rs8111085, but rs2288349 only has a much weaker LD with rs8111085 (r^2^ = 0.266). Therefore, it is plausible that the association with *H*.*pylori* infection is due to rs2288349 in a complete or perfect LD with unidentified causative SNPs. Further studies of *DNMT1* sequence variants and their biologic functions may shed light on associations of the *DNMT1* polymorphism and the risk for *H*.*pylori* infection and for gastric atrophy.

Over-expression of DNMT1 mRNA and protein is detected in gastric cancer suggesting that *DNMT1* may contribute to tumorgenesis [12], [35]. Moreover, our study showed *DNMT1* polymorphisms are not associated with gastric cancer, consistent with previous findings in which no correlation was found between the polymorphisms of the *DNMT1* gene and the risk for gastric cancer or colorectal cancer in the Iranian population [36], [37]. Conflicting results were reported in several association studies on polymorphisms of the *DNMT1* gene in relation to risks in breast cancer and ovarian cancer. While rs16999593 in exon 4 has been reported to be associated with an increased risk of sporadic infiltrating ductal breast carcinoma in Han Chinese female patients in one study [38], others found no apparent association between common DNMT1 polymorphisms or haplotypes and breast cancer risk among Chinese and European women [39]–[41]. The minor allele of rs9305012 at intron 23 of the *DNMT1* gene was found to be associated with a decreased risk for ovarian cancer among Caucasian multivitamin supplement users [42]. The difference in results from various studies may be due to different ethnic groups, unknown environmental factors or the interplay between environmental factors and genetic predisposition. Further studies are required to understand interactions of host and environmental factors and how and when these epigenetic interactions occur.

The limitation of our study is the small sample size for the *H.pylori* (+) cohort due to a low incidence in patients with gastric atrophy in China. A large-scale study recruiting more patients with gastric atrophy is required to confirm our findings in the future.

In conclusion, this study provides the first evidence of strong associations between polymorphisms and common haplotypes of the *DNMT1* gene with decreased risks for *H*.*pylori* infection in the Chinese population. In addition, polymorphisms of the *DNMT1* gene are also significantly associated with increased risks of developing gastric atrophy, suggesting that they could be used as biomarkers for predicting genetic susceptibilities to *H*.*pylori* infection and risk assessments in gastric atrophy. Further investigations are required to fully understand the biological importance of these polymorphisms.
